# The Importance and Scope of Remedies Increasing the Activity of the Lymphatics, with Special Reference to Iodalbin

**Published:** 1908-05

**Authors:** Noble M. Elerhart

**Affiliations:** Prof. & Head of Dep’t of Electro-therapy, Chicago College of Med. & Surgery; Prof. High-frequency & Vibration, Ill. School of Electro-therapeutics; Attending Surgeon, Frances Willard Hospital; formerly Attending physician Cook County Hospital


### THE IMPORTANCE AND SCOPE OF REMEDIES INCREASING THE ACTIVITY OF THE LYMPHATICS, WITH SPECIAL REFERENCE TO IODALBIN.

BY NOBLE M. EBERHART, M. S., M. D.

Prof. & Head of Dep't of Electro-therapy, Chicago College of Med. & Surgery; Prof. High-frequency & Vibration, Ill. School of Electro-therapeutics; Attending Surgeon, Frances Willard Hospital; formerly Attending physician Cook County Hospital.

In the treatment of diseased conditions a fact that is often overlooked or underestimated is the importance of the lymphatic system.

A great deal of stress is laid upon the blood and its components and upon various bodily functions without due consideration being given the role played by the lymphatics.

It is through the medium of the lymphatics that nutritive elements finally enter the blood and the tissues, and the waste products from the latter find their way out of the system.

It therefore follows that the successful treatment of any pathological condition will be materially aided by proper attention to the lymphatics, keeping them active and unobstructed.

Their importance from a therapeutic standpoint has received more consideration since the subject of mechanical vibratory stimulation has attained its present prominence, its results having been shown to rest, outside of stimulation of nerve centers, largely upon its effect as a lymphatic stimulant.

Aside from vibration, we have other useful means of acting upon the lymphatics, and omitting consideration of the various measures included under the head of physiological therapy, we come to the consideration of some of the medicinal preparations.

These were formerly known as alteratives and were supposed to influence the body in some mysterious way. It would seem, in the light of our present knowledge, that nearly all of these remedies are medicines which directly increase glandular activity and thus aid metabolic processes and increase the elimination of poisonous or waste products.

Among remedies which have stood forth prominently in this line, have been iodin and the iodides, the sulphides, phytolacca, etc.

Of these the one which has been used more than all others has been the iodide of potash. It has been uniformly satisfactory in results where its administration has been tolerated by the system and the principal drawback to its use in a much larger field has been the gastric irritation and the development of iodism, which so frequently follow its use.

It has therefore been restricted largely to use in secondary and tertiary syphilis, although it would otherwise be applicable to a wide range of diseases.

Under these conditions it is but natural that we should welcome any preparation which offers the opportunity of introducing iodin into the system in assimilable form and unaccompanied by unpleasant bye- or after effects. This is claimed to be the property of iodalbin.

Iodalbin is an almost tasteless, reddish powder, containing 21½ percent. of iodin in combination with albumen, thus being an iodin-proteid compound.

It does not dissolve in water, acids or alcohol, but is freely soluble in alkaline solutions.

Its insolubility in acid solutions ordinarily prevents breaking up in the stomach and consequently gastric irritation is rare.

When iodalbin reaches the alkaline intestinal secretions, it dissolves rather slowly and thus the absorption is slower than with iodide of potash, but since the iodin is in an organic combination it is much more thoroughly taken up and less passes out unappropriated.

In this manner the exaggerated effect following the rapid absorption of the alkaline iodides is obviated, but the final result is a more complete and thorough taking up by the system of the iodin which undoubtedly accounts for the fact that iodalbin with 21½ percent. of iodin apparently gives better results than iodide of potash with 76 percent. of iodin.

I have stated that iodalbin does not ordinarily produce gastric irritation. Granting that it remains stable in an acid medium, it would obviously have no opportunity of producing irritation of the stomach under ordinary conditions, thus being far superior to the alkaline iodides.

In two cases I have found that irritation of the stomach was undoubtedly produced by iodalbin, even when taken carefully as directed, an hour after each meal, followed by a full glass of water.

In giving this matter due consideration, it has occurred to me that in these cases where irritation is produced there must be necessarily a temporarily alkaline condition present in the stomach to admit of the breaking up of the compound and consequent absorption, and irritation of the gastric mucous membrane.

Under these circumstances, I would suggest that the stability of the iodalbin while in the stomach be insured by the adding of a few drops of hydrochloric acid to the glass of water with which the capsule is swallowed.

It stands to reason that any compound containing iodin may be taken in sufficient quantities, or under such circumstances that iodism may result; and that this does not occur frequently with iodalbin is a clinical fact which I attribute to a much better, though slower, assimilation of the iodalbin, so that the physiological effects do not ordinarily tend to become pathogenic.

The system can appropriate large quantities steadily administered before iodism occurs; while the sudden taking up of a large quantity will produce this symptom. Alkaline iodides being thoroughly soluble, the initial absorption is apt to be in the nature of a temporary overdose, and the balance passes out with the excrement entirely unabsorbed. Thus we sometimes have too great an effect with part of the dose and none whatever with the remainder. In iodalbin, we have slow, steady absorption of an organic compound, with its action always under control.

I have used iodalbin in specific conditions with uniformly satisfactory results, but in this article I am especially desirous of bringing out the point that it is not so much to be considered merely as a substitute for potassium iodide in syphilis, but that owing to its thorough assimilation and comparative lack of objectionable after or bye-effects, it gives opportunity for its use in a host of conditions where iodin is of advantage but many times unemployed. I refer to rheumatism, neuritis, asthma, arterial sclerosis, psoriasis, goitre, chronic bronchitis, anterior poliomyelitis and in obscure conditions where nutrition and elimination are interfered with without a precise diagnosis being easy or possible.

The usual dose is from 5 to 15 grains given an hour after each meal and preferably followed by a full glass of water. I have given 60 grains a day without producing untoward symptoms.

I have always used the 5 grain capsules for convenience.

As my special work is electro-and-radio-therapy, it is but fair to state that in most cases I have used physiological methods in conjunction with iodalbin. I give herewith a few cases selected from a number treated.

CASE I. Mrs. A. An artist working constantly at burning wood and leather, using a point heated after the manner of a Paquelin cautery but using wood alcohol in place of benzine as a fuel.

Breathing in the hot fumes of the wood and leather, at first made the lips dry and chapped and finally an intractable sore appeared on the lower lip which had existed for a number of weeks when I saw the case, and had resisted the local use of several washes and powders.

I was uncertain of the precise character of the sore, but continued the use of local antiseptics, applied the leucodescent light and vibrated cervical lymphatics, and cervical and dorsal spinal centers.

At the end of a month the improvement was very slight, but no additional symptoms had developed to give a possible clue to the diagnosis.

At this time I began the use of Iodalbin on the general hypothesis of increasing elimination through the lymphatics (which although slightly congested presented no marked enlargement or induration.)

I commenced with two capsules, an hour after each meal, followed by a full glass of water.

In a few days the sore began to heal rapidly and disappeared within two weeks.

CASE II. Mr. X. A case of locomotor ataxia receiving vibrations and high-frequency currents to improve locomotion and relieve the pains, gave evidence of the need of iodin for some manifestations of the specific origin of his present disease.

Prescribed two capsules an hour after each meal. Patient complained next day that his stomach had been entirely upset by one dose of the medicine. I did not think it possible and ascribed it to other causes and requested a further trial, using a single capsule with same results, so iodalbin was discontinued, as at this time the idea of using dilute hydrochloric acid had not occurred to me.

CASE III. Mr. Y. Tertiary syphilis with marked idiosyncrasy to potassium iodide.

I started with one capsule of iodalbin given an hour after each meal with a full glass of water. In one week I increased to two capsules and the third week to three capsules.

The condition of the patient rapidly improved with no indication of iodism until after several weeks on the full dose, when symptoms appeared, but subsided quickly on stopping the medicine.

This patient was under treatment a number of months and I found that one capsule three times a day for one week increasing to two the second week and three the third, maintaining the latter for three, four or five weeks, followed by a change to vegetable remedies (Echinacea, Baptisia and Phytolacca), for two or three weeks and then a return to the iodalbin kept the disease down to the mutual satisfaction of the patient and myself.

CASE IV. Mr. Z. Case of neuritis involving upper branches of anterior crural nerve, left side. The burning pain was very annoying and persistent.

Electrical and vibratory treatment afforded partial relief and on the addition of iodalbin, two capsules three times a day, marked improvement was noted.

CASE V. In a patient suffering from an old injury to the knee-joint, with fibrous ankylosis considerable pain of a rheumatic character was frequently present and persisted after the mobility of the joint had been restored. After the use of the salicylates and other remedies iodalbin was resorted to, one capsule three times a day, increasing in one week to two capsules.

The pain was somewhat relieved, but did not disappear and after three weeks the iodalbin was discontinued.

I cite this case because one must not expect to get uniformly successful results with every remedy and it is important that failures as well as successes be reported that the profession may not form an entirely one-sided view of any preparation or method.

CASE VI. Mr. R. Cnronic bronchitis with scanty expectoration. Two capsules of iodalbin three times a week produced quick and thoroughly satisfactory results.

A number of similar cases could be cited, and the remedy as I believe, especially satisfactory in these cases. The dose should be increased if necessary.

72 Madison street.

---

The seventy-sixth annual meeting of the British Medical Association will be held at Sheffield, in July.

---

Plans are outlined for the enlargement of the work of the Tabernacle Infirmary and Training School for Nurses, which is one of the departments of the Tabernacle system of institutional church work.

---

The Tennessee State Medical Association held its seventy-fifth annual meeting, at Knoxville, April 14, 15 and 16, 1908.

The following officers were elected: President, Dr. B. D. Bosworth, Knoxville; vice-president for east Tennessee, Dr. C. T. Carroll, Cleveland; vice-president for middle Tennessee, Dr. J. W. Brandau, Clarksville; vice-president for west Tennessee, Dr. W. T. Blanton, Union City; secretary, Dr. George H. Price, Nashville; treasurer, Dr. W. C. Bilbro, Murfreesboro; delegates to American Medical Association, Dr. S. W. Woodyard, Greenville; alternate, Dr. George R. West, Chattanooga, for 1908; for 1908-9, Dr. S. S. Crockett, Nashville; alternate, Dr. K. S. Howlett, Franklin. Next place of meeting, Nashville; time, April

